# Wildfire-Driven Shifts in Bird and Red Fox Activity: A Case Study from Biebrza National Park

**DOI:** 10.3390/biology14060685

**Published:** 2025-06-12

**Authors:** Jakub Gryz, Dagny Krauze-Gryz, Michał Brach

**Affiliations:** 1 Department of Forest Ecology, Forest Research Institute, Braci Leśnej 3, Sękocin Stary, 05-090 Raszyn, Poland; j.gryz@ibles.waw.pl; 2 Department of Forest Zoology and Wildlife Management, Institute of Forest Sciences, Warsaw University of Life Sciences WULS—SGGW, Nowoursynowska 159, 02-776 Warsaw, Poland; 3 Department of Geomatics and Land Management, Institute of Forest Sciences, Warsaw University of Life Sciences WULS—SGGW, Nowoursynowska 159, 02-776 Warsaw, Poland; michal_brach@sggw.edu.pl

**Keywords:** avian predators, rodent-eating raptors, scavengers, corvids, red fox, post-fire ecosystem recovery, post-fire foraging dynamics, seasonal predator activity

## Abstract

Climate change is expected to increase the frequency and severity of wetland fires, posing risks to biodiversity. We investigated changes in the foraging activity of predators and scavengers in response to a large wildfire (over 5500 ha of marshland) in Biebrza National Park, NE Poland, in the spring of 2020. Using GLMMs on bird records from 10 points in burned and unburned areas across seasons, we assessed the responses of rodent-eating raptors, corvids, and wading birds (herons, cranes, and storks). Bird numbers varied significantly with the interaction between fire and season, although fire alone was not a significant factor. Rodent-eating owls and raptors were recorded most frequently in burned areas during summer 2020 (3.5 in burned vs. 1.7 records/hour in control areas), likely due to improved hunting conditions in reduced vegetation. Corvids peaked in burned areas just after the fire (3.1 vs. 1.4 records/hour), presumably in response to carrion availability. Wading birds showed a slight post-fire increase in burned habitats (2.4 vs. 1.7 records/hour), yet their abundance was mostly season-driven. These results suggest that fire-altered habitats may provide short-term foraging advantages for certain bird groups. However, responses are species-specific and depend on fire timing, intensity, and habitat preferences.

## Simple Summary

Climate change is likely to cause more frequent and intense wetland fires, which can affect wildlife. This study looked at how birds that eat rodents or scavenge reacted to a large wildfire in Biebrza National Park, Poland, in 2020. We observed bird activity (expressed by the number of observed birds during a 1-h period) in both burned and unburned areas over different seasons. We found that bird activity depended on the combination of two factors, i.e., the fire and the season. Rodent-eating raptors and owls were more active in burned areas during summer, likely because it was easier to hunt when plant cover was removed by fire. Scavenging corvids were most active in burned areas right after the fire, probably due to the presence of dead animals and good feeding opportunities. Activity of wading birds (herons, cranes, and storks) feeding on a variety of food items (both plant and animal food) increased slightly in burned areas, but their activity mostly followed seasonal patterns. Overall, some bird species may benefit temporarily from fire-damaged areas, but their responses vary depending on the species and conditions of the fire.

## 1. Introduction

Fires of natural or anthropogenic origin shape some ecosystems on Earth; this disturbance can maintain the landscape and influence many processes like vegetation structure, carbon, and hydrological cycle, climate, and others [1,2,3]. Because of climate change, wildfires occur more frequently and can have a devastating impact on wildlife, humans, and the economy [3,4,5,6]. Wetlands are recognized as biodiversity hotspots for both plants and animals [7,8], and Central European wetlands seem to be the most threatened by changes to the hydrological regime as predicted by climate projections [9]. Although burning can be among conservation measures within management strategies of marshlands [10], the predicted droughts affecting wetland water regimes are likely to result in more frequent fires [11]. Their effect, especially on more sensitive species, is hard to predict as the impact of wildfires on birds and mammals is multiform and insufficiently known. It may depend on fire characteristics, species’ traits [12], and the time scale considered [13]. First, wildfire can directly influence animals by killing or injuring them [14,15,16,17]. Also, reduced protective cover may result in higher predation rates of survivors and newcomers in post-fire areas [18,19,20]. An increased predation risk can also be due to reduced prey body condition after fire and/or an increased need to forage in risky areas due to being energetically stressed [21,22]. All these may create good feeding opportunities for predators and scavengers. Indeed, corvids and some predatory birds travel to fire-affected areas to take advantage of easier hunting in burnt landscapes and increased scavenging opportunities [22,23,24]. They may even exploit fire fronts to capture prey flushed out by flames [23]. Adversely, fire may affect the rodent community [25], thus reducing prey for rodent-preying predators, which may result in significant declines in predatory species [13].

The Biebrza Valley is recognized as one of the most extensive wetlands in central and western Europe. In this study, we evaluated the impact of the large wildfire of spring 2020 (the largest open landscape fire in Poland after World War II, [26]) on the foraging activity of different groups of predators and scavengers. Following the expected increase in fire frequency, more knowledge of the impact on freshwater wetlands is crucial for future risk assessment. We focused on birds that belong to different trophic groups (and can be classified as predators, scavengers, or omnivores) by comparing the intensity of their penetration of open habitats of a vast freshwater wetland area shortly after the fire and during the following seasons. We may assume that in the case of rodent-eating avian predators, fire must have sharply reduced the abundance of available prey, especially in the short term [13,25]. Therefore, their reduced activity in the burned areas was expected, with an increased foraging activity when the area starts to be recolonized [19,20,21]. In turn, corvids, taking advantage of the increased availability of carrion of fire victims [24], were expected to be more active in the fire-affected areas shortly after the fire event. We also focused on wetland-associated long-legged wading birds that prey on variable vertebrate and invertebrate prey. We assumed that areas heavily disturbed by fire, with a limited vegetation cover, can be attractive foraging sites for this diverse bird group [13,27]. Finally, we focused on the red fox *Vulpes vulpes*, the most common and widely distributed medium-sized carnivore [28,29,30,31] for which small rodents are crucial prey [32,33]. Small rodent availability shapes space use by the red fox [30]. Although the species richness and abundance of small mammals are often lowest immediately after a fire, they recover (or even surpass pre-fire levels) as vegetation progresses [34]. We, thus, predicted that in winter (approximately eight months after the fire), small mammal availability would be similar in the fire-disturbed and the control areas, reflected by the similar penetration intensity of this medium-sized carnivore.

## 2. Methods

### 2.1. Study Area

**Figure 1:**
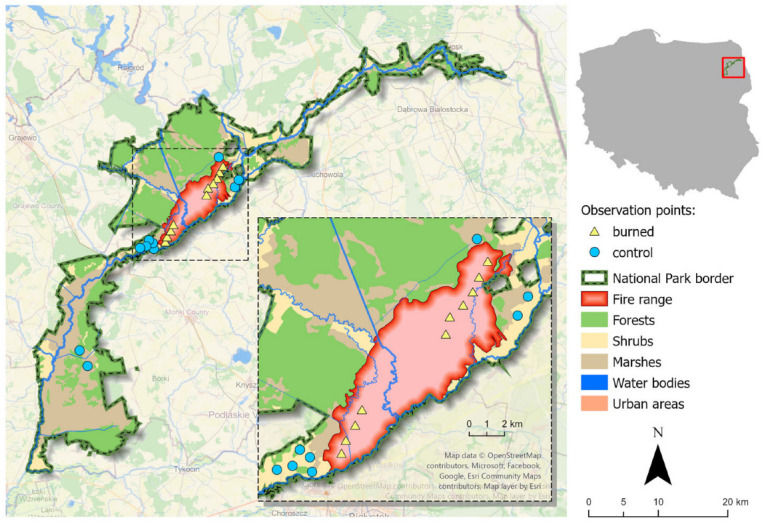
Location of Biebrza National Park in Poland, range of the wildfire in the Biebrza National Park (19–26 April 2020), and location of bird observation points within the burned and outside burned area (control).

The study was conducted in Biebrza National Park, located in NE Poland (53°28′48.3″ N 22°38′19.3″ E, Figure 1). It is the largest (nearly 60,000 ha) national park in Poland and one of the biggest in Europe. It is one of central and western Europe’s biggest, still well-preserved wetland areas [35]. The most important element of this area is typically lowland, around 160 km long, and meandering the Biebrza River. Biebrza is one of central and western Europe’s last big natural rivers. The national park’s landscape is dominated by open meadows, pastures, and rushes. Over 40% of habitats are hydrogenic [36]. Forests constitute around 25% of the park and are dominated by birches *Betula* spp., black alder *Alnus glutinosa*, and Scots pine *Pinus sylvestris* [37]. Biebrza National Park is recognized as a Natura 2000 site and included in the List of Wetlands of International Importance [38]. Because of climate change, flooding in the Biebrza River has been highly limited in recent decades [39]. Historically, all areas were flooded in spring, but recently, permanent drought has been noted. As a result, wildfires are becoming more and more common [40]. Most of the fires in the study area occur due to illegal burning [30].

### 2.2. The Fire in the Biebrza National Park

**Figure 2:**
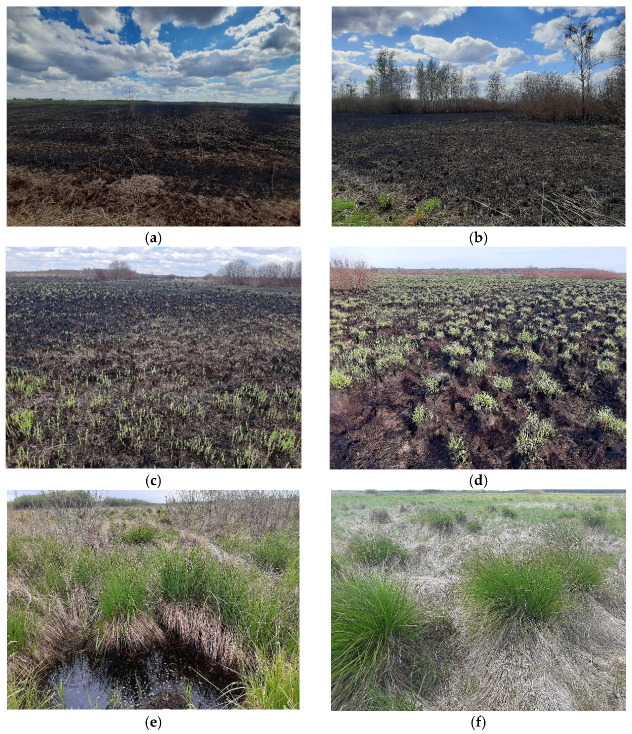
Landscape of study area in burned and control sites: (**a**,**b**) burned area just after the fire, (**c**) burned area—regenerating vegetation on 4 May 2020, (**d**) burned area—regenerating vegetation on 13 May 2020, and (**e**,**f**)—unburned area on 25 May 2020.

In April 2020, the water level in the river was the lowest (104 cm) compared to all measurements performed in this month since 1950, when the measurements started [40]. The wildfire that broke out on 19 April (and lasted until 26 April) was the biggest ever recorded in this area. Over 5526 ha of meadows and forests burned (Figure 1). The very rapid spread of the fire in the open space, with the blowing wind changing directions and the inaccessibility of the area for fire-fighting vehicles, made the fire one of the most extensive wildfires in Poland after the Second World War and the biggest outside forests [30]. Nevertheless, the fire did not affect the peat substrate. Detailed vegetation cover data were unavailable. Nevertheless, in spring 2020, burned and controlled areas differed very clearly; in summer 2020, this difference was less vivid, yet the vegetation in the burned areas was less abundant [41] (Figure 2). Vegetation seemed to recover within a few months after the disturbance [42], and in spring 2021, the vegetation did not differ visibly (Figure 2).

### 2.3. Fieldwork

#### 2.3.1. Assessment of the Number of Fire Victims

Just after the end of the fire, we counted all dead vertebrates on a 23,438 m long and 4 m wide transect (93,752 m^2^ in total, around 0.2% of the fire-destroyed area) to assess carrion availability for predators and scavengers and to roughly estimate the number of fire victims. We penetrated different parts of the burned area, moving along the selected observation points (see below). An observer walked slowly, looking for even the smallest vertebrates on the right and left sides of the transect. We used a GPS receiver and a 2 m long stick to assess the transect width properly. In the case of remnants of bird nests, we usually could not assess if they were occupied and how many chicks died/eggs were destroyed. Thus, to avoid overestimating the victims, we arbitrarily claimed that in one nest, one bird died. Finally, we extrapolated our results on the entire burned area to roughly estimate how many vertebrates were fire victims. This data gave us information about the potential scavenger food base and the scale of loss for predators that hunt small mammals. During the fieldwork, we omitted remnants of animals that had clearly died before the fire, i.e., if we found only bones from big mammals without other tissues. In such cases, we assumed that they had probably been killed by wolves *Canis lupus*, or, in the case of wild boars *Sus scrofa*, were victims of African swine fever that broke out in this part of Poland in 2014 [43,44].

#### 2.3.2. Assessment of Bird Activity in Burned and Unburned Areas

Simultaneously, we selected 10 bird observation points in the burned area. The distance between the nearest points was around 1 km. Similarly, 10 adequate observation points were selected in unburned (i.e., control) areas of the National Park around (Figure 1). The points were set as systematically as possible. Yet, their placement was adjusted so as to place them in similar surroundings (i.e., not in arable lands) and to avoid forest and willow bushes, which could hinder visibility and limit observational distance. Point placement was also affected by logistical reasons, as some parts of the park were inaccessible (i.e., there were no roads in the vicinity, and the interior of the marshes had very few paths and was hard to penetrate due to extensive peat bogs, marshes, and floodplains, being able to be reached only by boat when flooded). Each point was visited twice in spring (just after the fire, i.e., at the very beginning of May), in summer (July), and autumn (November) of 2020, and the next spring, in early May of 2021. One observation session at one point lasted 30 min. After this time, the observer moved to another point. The procedure was conducted for a few consecutive days. We pooled observations from one point from two 30-min sessions in the season. As a result, observation was conducted for 1 h each season at each point. Altogether, we conducted the observations for 80 h. Observations were conducted by binocular ZEISS (Oberkochen, Germany) 10 × 42 in the morning (mostly between 6 and 10 a.m.) during good weather conditions. We recorded the number of birds assigned to a certain species observed during one session. If one bird was observed for a longer period during a single session, it was counted just once. If a bird appeared more than twice during a session and we were not sure if it was the same individual or a new one, we adopted a 15-min time lapse until we recorded it once more. We pooled observations of various bird species into four groups: (i) raptors and owls preying on small rodents (the common buzzard *Buteo buteo* [45,46], rough-legged buzzard *Buteo lagopus,* the common kestrel *Falco tinnunculus* [47], long-eared and short-eared owl *Asio otus* and *A. flammeus* [48,49], lesser spotted eagle *Clanga pomarine* [50], greater spotted eagle *Clanga clanga* [51], the western marsh harrier *Circus aeruginosus*, or the Montagu’s harrier *Circus pygargus* [52,53]), (ii) corvids (i.e., the common raven *Corvus corax*, hooded crow *Corvus cornix*, and magpie *Pica pica*), being opportunistic foragers often feeding on carrion, [54,55,56,57,58,59]), and (iii) long-legged wading birds (i.e., grey heron *Ardea cinerea*, great egret *A. alba*, white and black storks *Ciconia ciconia*, *C. nigra*, crane *Grus grus*), opportunistic predators, eating various prey [60,61,62,63,64]. From our observations, we excluded birds from the last group that flew at high altitudes, and their presence was clearly unrelated to foraging or prey search. The remaining raptors were other raptor species (i.e., white-tailed eagle *Haliaeetus albicilla*, goshawk *Astur gentilis*, sparrowhawk *Accipiter nissus*, hobby *Falco subbuteo*).

#### 2.3.3. Penetration Intensity of Burned and Unburned Areas by the Red Fox

During winter 2020/2021 (approx. 10 months after the fire), we conducted snow tracking of the red fox in burned and unburned areas. The method of snow tracking is a well-established method for studying red fox habitat use, and it estimates activity levels of this medium-sized carnivore [29,31]. We carried out snow tracking 1–3 days after a snowfall, adjusting the number of tracks registered for 24 h of snow cover. The snow tracking in the burned and control areas was conducted during the same days, so the snow conditions were similar. We calculated a relative index of fox abundance as N tracks/km/24 h of snow cover. Tracks were identified based on shape, size, and the placement of toepads. The length of the transects in the burned area was 22 km, and in the reference area, 23 km. The transects were placed in open areas, also in the vicinity of the birds’ observation points.

### 2.4. Statistical Analysis

The difference in the total number of birds recorded in burned and control areas was checked with student’s *t*-tests. To test if the number of recorded birds assigned to the four groups changed in response to the fire occurrence (and its interaction with season), we used two-way PERMANOVA (999 permutations) with repeated measures, using Bray–Curtis dissimilarity. The obtained *p*-values were adjusted for multiple comparisons using Bonferroni correction. Fire occurrence (burned vs. control areas), season (spring 2020—just after the fire, summer 2020, autumn 2020, or spring 2021), and the interaction between the two were included as independent variables. This analysis was conducted using the adonis2() function from the vegan package [65] in R [66]. Next, for each of the four groups of birds, we used a generalized linear mixed model (GLMM) to check if fire occurrence or the interaction between fire occurrence and the season affected the number of records of each defined bird group separately. The models assumed a Poisson distribution with a log link function. The response variable was the number of birds recorded per observation unit. Two fixed factors were included: fire (burned vs. control) and its interaction with season (spring_2020, summer_2020, autumn_2020, and spring_2021). A random effect was specified for the sampling location (observation points) to account for repeated measures. Next, post hoc pairwise comparisons using estimated marginal means (Bonferroni corrected *p* values) were used to test the difference between burned and control areas with seasons. Mann–Whitney U test was used to compare the number of red fox tracks in the burned and control areas. The analyses were completed with SPSS (PS IMAGO PRO 10) software [67].

## 3. Results

### 3.1. Number of Fire Victims

We found 47 fire victims on the transect in the burned area. The biggest share of them were amphibians (N = 19). The biggest animals found dead were red fox and white stork (Table 1). Extrapolating our results, on the whole, burned area, we can roughly assess the minimum number of vertebrates killed by fire on 27,630 individuals.

### 3.2. Bird Activity in the Burned and Unburned Areas

Overall, we recorded 531 bird observations, 272 in the burned and 259 in the reference areas; on average, there were 6.8 birds per hour of observations in the burned (SE = 0.57) and 6.48 (SE = 0.46) in the control sites (Appendix A). The number of observed birds assigned to the four groups (Table 2) depended on the interaction between the seasons and the fire occurrence (two-way PERMANOVA with repeated measures, fire*season F = 0.071, df = 3, *p* < 0.05).

**Figure 3:**
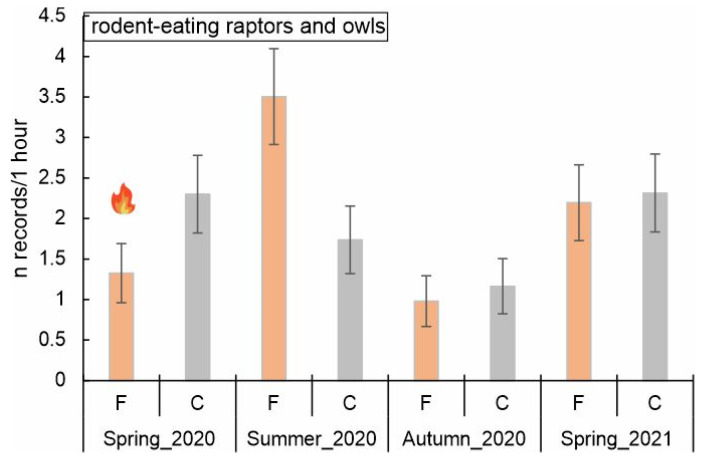
Predicted number of records of rodent-eating owls and raptors (marginal means, ±SE) across different seasons (spring_2020, i.e., just after the fire; marked with flame symbol) and fire conditions (F—burned, C—control areas). Post hoc pairwise comparisons (Bonferroni corrected) showed the difference between burned and control areas (*p* = 0.017) in summer 2020.

Owls and raptors that prey on rodents were recorded 76 times in burned and 72 times in control areas (Table 2). The overall model (GLMM) for rodent-eating raptors and owls was statistically significant (F = 3.259, df = 7, *p* = 0.005). The interaction between fire and season was significant (F = 3.668, df = 6, *p* < 0.005), indicating that the effect of fire changed seasonally. Nevertheless, fire’s main effect was insignificant (F = 0.111, df = 1, *p* > 0.05). Rodent-eating raptors and owls were recorded most frequently in burned areas in summer 2020 (a few months after the fire, 3.5 records/hour vs. 1.7 in control points at the same time, Figure 3). In turn, in spring 2020 (just after the fire) the abundance of birds was lower in burned than in control points (1.3 vs. 2.2 records/hour, Figure 3). Tested interactions pointed to a substantial increase (2.64-fold) in rodent-eating raptor abundance in burned plots in summer 2020 (β = 0.972, SE = 0.322, *p* = 0.004) (Table 3). Other interactions were not significant (Table 3). Post hoc pairwise comparisons of estimated marginal means revealed a significant difference between burned and control areas only in summer 2020 (*p* = 0.017) (Figure 3).

**Figure 4:**
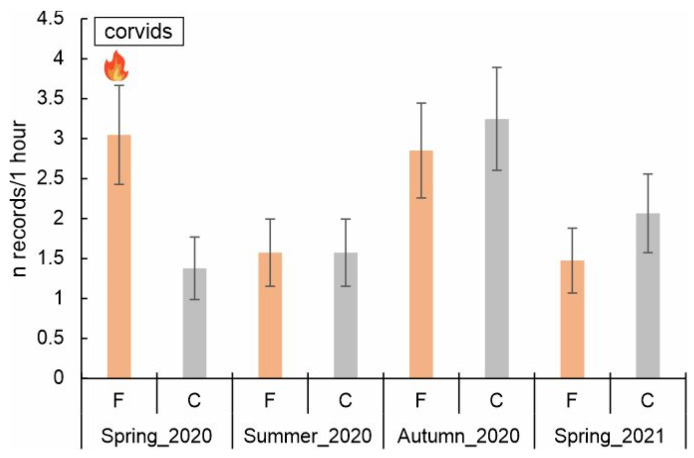
Predicted number of records of corvids (marginal means, ±SE) across different seasons (spring_2020, i.e., just after the fire; marked with flame symbol) and fire conditions (F—burned, C—control areas). Post hoc pairwise comparisons (Bonferroni corrected) showed differences (*p* = 0.025) between burned and control areas in spring 2020.

Corvids were recorded 175 times, 91 times in burned areas and 84 times in the control areas. The number of observed corvids in the burned and control areas was similar during three out of four seasons. Only shortly after the fire (in spring 2020), the number of observations in burned areas was higher than in control points (3.1 vs. 1.4 records/hour). The model for corvids (GLMM) was statistically significant (F = 2.727, df = 7, *p* = 0.014). The effect of fire on corvid abundance depended on the season (Fire*Season, F = 3.159, df = 6, *p* = 0.008), while the fire occurrence itself was not significant (*p* > 0.05). In spring 2020 (just after the fire), corvid abundance was more than twofold higher in burned than in control observation points (β = 0.794, *p* = 0.026). In turn, negative interactions were shown for summer 2020 (β = −0.661, *p* = 0.035) and spring 2021 (β = −0.726, *p* = 0.024), pointing to a lower abundance of corvids in burned plots at those times than in spring 2020 (just after the fire). The abundance of corvids in the control areas was highest in autumn 2020 (β = 0.857, *p* = 0.009) (Table 4, Figure 4). Post hoc pairwise comparisons showed the effect of fire occurrence on corvids’ abundance in spring 2020 (*p* = 0.025) (Figure 4).

**Figure 5:**
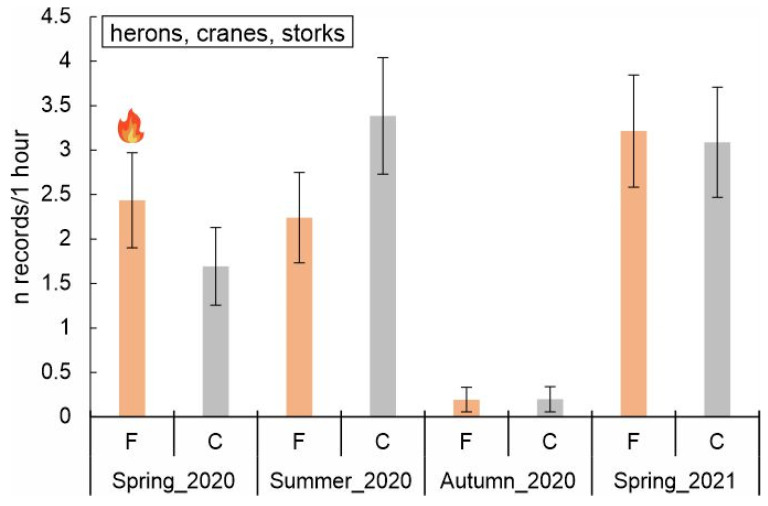
Predicted number of records of wading birds (herons, cranes, and storks) (marginal means, ±SE) across different seasons (spring_2020, i.e., just after the fire; marked with flame symbol) and fire conditions (F—burned, C—control areas). No significant differences between burned and control plots in any season were shown by post hoc pairwise comparisons.

The long-legged wading birds (herons, cranes, and storks) were observed 167 times, 83 and 84 times in burned and control areas, respectively. The overall model for wading birds was highly significant (F = 5.010, df = 7, *p* < 0.001), indicating that the predictors (fire, season, and their interaction) explained a substantial amount of variance in wading bird abundance. The main effect of fire was not significant (*p* = 0.980). However, the interaction between fire and season was highly significant (F = 5.830, df = 6, *p* < 0.001). In burned plots, a substantial reduction in wading birds’ abundance was observed in autumn 2020 (β = –2.526, *p* < 0.001). Within control plots, abundance varied seasonally. Wading birds’ abundance was significantly higher in summer 2020 and marginally higher in spring 2021, while a significant decrease was observed in autumn 2020 (Table 5, Figure 5). Post hoc comparisons of estimated marginal means showed no statistical differences between burned and control areas in any season.

The overall model (GLMM) for the remaining birds of prey pooled together was not significant (F = 0.784, df = 7, *p* > 0.05); their abundance was not affected by either the fire occurrence (F = 0.005, df = 1, *p* > 0.05) or the interaction between the two factors (F = 0.836, df = 6, *p* > 0.05).

### 3.3. Penetration Intensity of Burned and Unburned Areas by the Red Fox

The red fox density index during the winter of 2000/2021 was 6.86 tracks/km/24 h (SE = 0.411) in the burned area and 6.13 tracks/km/24 h (SE = 0.432) in the control area. These values did not differ significantly (Mann–Whitney test, U = 1851, *p* > 0.05).

## 4. Discussion

### 4.1. Fire-Induced Mortality

Our knowledge of fire-induced mortality of animals, especially via high-intensity wildfires as opposed to planned fires, is very low [21]. Our estimations of 30,000 fire victims are probably highly understated, as spotting all small vertebrates in black ash is impossible, or their bodies may have been completely calcined [20]. Also, rodents, moles *Talpa europaea*, and some reptiles could have died in burrows because of high temperatures, lack of oxygen, or toxic smoke [20]. Some other individuals could have died even later due to, e.g., fire injuries [21]. It is unclear how many animals may have survived the fire [20,21], yet we can assume that a big part of the small mammals present in this area probably died during or after the fire. The most abundant rodent species in Biebrza Marshes is the root vole *Microtus oeconomus*. The spring density of this population fluctuates yearly, from just a few to 150 ind./ha [68]. Its abundance in the burned areas before the fire occurred is unknown, yet the population abundance in other Biebrza marshes areas was over 50 ind./ha [41]. Amphibian density on open marshland is challenging to estimate. However, on sedgeland in the Narew River Valley (located nearby, Biebrza being its tributary), the moor frog *Rana arvalis* density was between 300 and 1800 ind./ha. These values may be even higher in riparian habitats along the river [69]. Considering the annual life cycle of anurans inhabiting our study area, all the species of frogs and toads must have already emerged from the hibernation sites at the time of the fire, thus being at risk of fire. Even if just a fraction of amphibians were killed by the fire, the number of fire victims would be over 1.5 million. Overall, an estimated animal mortality would result in a very high short-term carrion availability for scavengers.

### 4.2. Bird Activity as a Response to Fire Occurrence

The two factors affecting bird occurrence in the burned areas are vegetation and food alterations [17]. The effect of fire is vivid primarily shortly after the event [70] and is species dependent [17]. In our case, a higher abundance of corvids in the burned area was observed shortly after the fire. This aligns with our data about available carrion assessment and the nature of the three observed corvid species, i.e., raven, hooded crow, and magpie, as opportunistic feeders [54,55,56,57,58,59]. During further observation sessions (i.e., seasons), the number of records of corvids in burned and control areas was similar, suggesting that trophic conditions were no longer affected by the fire event.

The fire probably reduced small rodent availability in the short term [17,29,41]. Indeed, in summer 2020, vole density was much lower in burned than in fire-unaffected areas [41]. However, at that time, the number of records of rodent-eating raptors peaked in fire-disturbed areas and was much higher than in control points. The difference in fresh vegetation matter between burned and unburned areas was not detectable on satellite images just 2 months after the fire [42], so small mammals possibly started recolonizing this site. Still, the vegetation cover was much reduced (like clumps of sedge or accumulated dry plant matter) compared to control areas, offering less cover for small mammals [41]. Therefore, rodents that colonized areas were probably easy prey for raptors [22].

The abundance of herons, cranes, and storks changed with the season and was very low in autumn when these birds had already flown away to their wintering sites [71,72,73,74] and highest in spring 2021 (the year after the fire, when the water level was high). They were more commonly observed in the burned areas than in the control areas shortly after the fire, and more often observed in control sites in summer. Yet these differences were insignificant. The burned area could have posed an attractive foraging site because of the presence of carrion and wounded animals, and its easy access to invertebrates with the scarce vegetation cover [17].

### 4.3. Response to Fire Occurrence by the Red Fox

Fire can influence predator–prey interactions by rapidly modifying the distribution of concealment cover and food resources [75]. Our results documented no differences in the red fox density index in the burned and control areas in winter. Unfortunately, we have no data on food availability in the two areas at that time. Nevertheless, the most vivid changes (decrease in the number of rodents and increase in the carrion availability) were expected shortly after the fire (i.e., in spring and summer). Also, vegetation cover alteration, enabling easy hunting, was most significant at that time ([41,42], own observations). Probably, almost 10 months after the fire, prey availability for foxes was similar in burned and unburned habitats. Seemingly, the response to fire by medium-sized carnivores may vary and be context-dependent (review in [23]). In general, and according to the literature, the red fox was shown to respond positively to fire. However, some studies showed a neutral response [76]. For example, in Australia, fire history did not significantly affect red fox distribution at either landscape or site scales [77].

## 5. Conclusions

Large-scale wildfire in the Biebrza National Park locally sharply reduced populations of small vertebrates. This study highlights wildfire’s immediate and short-term ecological consequences on bird activity and predator dynamics in a marshland ecosystem of Biebrza National Park. Although direct estimations of fire-induced animal mortality are limited, the available evidence suggests a substantial impact, particularly on small vertebrates. This supported elevated scavenger (i.e., corvids) activity due to increased carrion availability in burned areas shortly after the fire. Also, other predators (i.e., rodent-eating birds of prey and owls) responded positively to the fire event, taking advantage of the process of recolonization of burned areas by small mammals that occurred a few months after the fire, albeit with increased vulnerability to predation due to diminished vegetation cover. Conversely, red foxes exhibited no significant differences in winter activity between burned and unburned areas, suggesting that prey availability and habitat structure had equilibrated by that time. Overall, while the wildfire caused notable short-term ecological changes, these effects weakened within a year, underscoring the resilience of the studied marshland ecosystem. However, climate change results in increased wildfire frequency and intensity. While fire-adapted species may be less vulnerable to fire events, changes in the fire regime and their intensity may make many other species or areas fire-affected [21]. Future research should aim to quantify longer-term consequences and further explore the nuanced responses of different trophic groups to fire disturbances.

## Figures and Tables

**Table 1:** List of fire victims detected on the sample transect (23,438 m long, 4 m wide) in the burned area of Biebrza National Park.

Fire Victim	N
*Lepus europaeus* (juv.)	1
*Erinaceus* sp.	1
*Vulpes vulpes*	1
*Talpa europaea*	2
*Sorex* sp.	1
Small rodents unidentified	5
*Pica pica*	1
*Ciconia ciconia*	1
Bird unidentified	3
*Bufo* spp.	9
Ranidae	6
*Triturus*/*Lissotriton*	1
Amphibian unident.	3
*Anguis fragilis*	1
*Natrix natrix*	1
*Lacerta*/*Zootoca*	1
Fish	3
Unidentified vertebrates (excl. birds)	2
bird nests	4
Total	47

**Table 2:** The number of birds observed from different trophic groups in the burned (F) and control (C) areas in the Biebrza National Park during four seasons. Spring 2020—just after the fire was extinguished. Wading birds—herons, cranes, and storks.

Season		Birds’ Group (N Records)				
		Rodent-Eating Raptors and Owls	Corvids	Wading Birds	Other Raptors	Total
Spring 2020	F	13	31	25	5	74
	C	22	14	17	8	61
Summer 2020	F	35	16	23	6	80
	C	17	16	34	3	70
Autumn 2020	F	7	29	2	6	44
	C	10	33	2	2	47
Spring 2021	F	21	15	33	5	74
	C	23	21	31	6	81

**Table 3:** The effect of fire occurrence, season (spring_2020, i.e., just after the fire) and the subsequent periods), and their interaction on the number of records of rodent-eating raptors (n records/1 h) observed in the 10 sampling locations (observation plots) located within the burned area and in the control areas shown by GLMM. 0 *—reference category. Significant effects marked in bold.

Model Term	β	SE	t	*p*	95% CI	
					Lower	Upper
Intercept	0.833	0.2085	3.997	<0.001	0.418	1.249
Fire = fire	−0.551	0.3448	−1.599	0.114	−1.239	0.136
Fire = control	0 *					
**[Fire = fire] × [Season = Summer_2020]**	**0.972**	**0.3224**	**3.015**	**0.004**	**0.329**	**1.615**
[Fire = fire] × [Season = Autumn_2020]	−0.301	0.4212	−0.715	0.477	−1.141	0.538
[Fire = fire] × [Season = Spring_2021]	0.504	0.3478	1.450	0.151	−0.189	1.198
[Fire = fire] × [Season = Spring_2020]	0 *					
[Fire = control] × [Season = Summer_2020]	−0.281	0.3179	−0.885	0.379	−0.915	0.352
[Fire = control] × [Season = Autumn_2020]	−0.681	0.3597	−1.894	0.062	−1.398	0.036
[Fire = control] × [Season = Spring_2021]	0.006	0.2944	0.021	0.983	−0.581	0.593
[Fire = control] × [Season = Spring_2020]	0 *					

**Table 4:** The effect of fire occurrence, season (spring_2020, i.e., just after the fire), and the subsequent periods, and their interaction on the number of records of corvids (n records/1 h) observed in the 10 sampling locations (observation plots) located within the burned area and in the control areas shown by GLMM. 0 *—reference category. Significant effects marked in bold.

Model Term	β	SE	t	*p*	95% CI	
					Lower	Upper
Intercept	0.320	0.2835	1.130	0.262	−0.245	0.885
**Fire = fire**	**0.794**	**0.3486**	**2.277**	**0.026**	**0.099**	**1.489**
Fire = control	0 *					
**[Fire = fire] × [Season = Summer_2020]**	**−0.661**	**0.3078**	**−2.149**	**0.035**	**−1.275**	**−0.048**
[Fire = fire] × [Season = Autumn_2020]	−0.067	0.2583	−0.258	0.797	−0.582	0.448
**[Fire = fire] × [Season = Spring_2021]**	**−0.726**	**0.3145**	**−2.308**	**0.024**	**−1.353**	**−0.099**
[Fire = fire] × [Season = Spring_2020]	0 *					
[Fire = control] × [Season = Summer_2020]	0.134	0.3660	0.365	0.716	−0.596	0.863
**[Fire = control] × [Season = Autumn_2020]**	**0.857**	**0.3190**	**2.688**	**0.009**	**0.222**	**1.493**
[Fire = control] × [Season = Spring_2021]	0.405	0.3450	1.175	0.244	−0.282	1.093
[Fire = control] × [Season = Spring_2020]	0 *					

**Table 5:** The effect of fire occurrence, season (spring_2020, i.e., just after the fire) and the subsequent periods), and their interaction on the number of records of herons, cranes, and storks (n records/1 h) observed in the 10 sampling locations (observation plots) located within the burned area and in the control areas shown by GLMM. 0 *—reference category. Significant effects marked in bold.

Model Term	β	SE	t	*p*	95% CI	
					Lower	Upper
Intercept	0.526	0.2585	2.037	0.045	0.011	1.042
Fire = fire	0.364	0.3392	1.072	0.287	−0.313	1.040
Fire = control	0 *					
[Fire = fire] × [Season = Summer_2020]	−0.083	0.2889	−0.289	0.774	−0.659	0.493
**[Fire = fire] × [Season = Autumn_2020]**	**−2.526**	**0.7348**	**−3.437**	**<0.001**	**−3.991**	**−1.061**
[Fire = fire] × [Season = Spring_2021]	0.278	0.2651	1.047	0.299	−0.251	0.806
[Fire = fire] × [Season = Spring_2020]	0 *					
**[Fire = control] × [Season = Summer_2020]**	**0.693**	**0.2970**	**2.333**	**0.022**	**0.101**	**1.285**
**[Fire = control] × [Season = Autumn_2020]**	**−2.140**	**0.7475**	**−2.863**	**0.005**	**−3.630**	**−0.650**
[Fire = control] × [Season = Spring_2021]	0.601	0.3018	1.991	0.050	−0.001	1.202
[Fire = control] × [Season = Spring_2020]	0 *					

## Data Availability

The original contributions presented in this study are included in the article. Further inquiries can be directed to the corresponding author.

## Appendix A

**Table A1:** Number of records of birds assigned to each group per 1 h observation.

Point_ID	Fire Event	Season	Rodent-Eating Raptors and Owls	Wading Birds	Corvids	Other Raptors
1	fire	2020 spring	3	2	4	0
2	fire	2020 spring	1	3	2	1
3	fire	2020 spring	1	3	3	2
4	fire	2020 spring	1	1	3	0
5	fire	2020 spring	1	1	3	0
6	fire	2020 spring	0	2	3	0
7	fire	2020 spring	2	5	1	1
8	fire	2020 spring	1	2	3	1
9	fire	2020 spring	2	4	6	0
10	fire	2020 spring	1	2	3	0
11	control	2020 spring	1	2	2	0
12	control	2020 spring	3	2	2	0
13	control	2020 spring	1	1	0	1
14	control	2020 spring	6	0	1	0
15	control	2020 spring	3	1	1	4
16	control	2020 spring	4	2	0	0
17	control	2020 spring	2	2	1	0
18	control	2020 spring	0	2	1	2
19	control	2020 spring	1	4	1	0
20	control	2020 spring	1	1	5	1
1	fire	2020 summer	5	3	2	1
2	fire	2020 summer	5	2	1	1
3	fire	2020 summer	3	1	4	2
4	fire	2020 summer	3	2	0	1
5	fire	2020 summer	3	2	2	1
6	fire	2020 summer	2	3	1	0
7	fire	2020 summer	4	6	2	0
8	fire	2020 summer	2	4	2	0
9	fire	2020 summer	5	0	2	0
10	fire	2020 summer	3	0	0	0
11	control	2020 summer	3	4	3	1
12	control	2020 summer	2	2	1	0
13	control	2020 summer	1	5	1	1
14	control	2020 summer	0	1	1	0
15	control	2020 summer	1	3	1	0
16	control	2020 summer	2	4	2	0
17	control	2020 summer	3	5	1	1
18	control	2020 summer	1	3	3	0
19	control	2020 summer	3	3	2	0
20	control	2020 summer	1	4	1	0
1	fire	2020 autumn	2	1	4	1
2	fire	2020 autumn	0	0	1	0
3	fire	2020 autumn	2	0	2	0
4	fire	2020 autumn	0	0	0	1
5	fire	2020 autumn	0	1	1	0
6	fire	2020 autumn	2	0	2	1
7	fire	2020 autumn	0	0	3	1
8	fire	2020 autumn	0	0	9	1
9	fire	2020 autumn	1	0	6	1
10	fire	2020 autumn	0	0	1	0
11	control	2020 autumn	3	1	0	1
12	control	2020 autumn	2	0	1	1
13	control	2020 autumn	0	0	2	0
14	control	2020 autumn	1	1	8	0
15	control	2020 autumn	0	0	9	0
16	control	2020 autumn	1	0	4	0
17	control	2020 autumn	1	0	0	0
18	control	2020 autumn	0	0	5	0
19	control	2020 autumn	0	0	2	0
20	control	2020 autumn	2	0	2	0
1	fire	2021 spring	5	3	2	1
2	fire	2021 spring	1	1	3	0
3	fire	2021 spring	2	2	2	1
4	fire	2021 spring	5	2	1	1
5	fire	2021 spring	1	2	1	0
6	fire	2021 spring	1	1	0	0
7	fire	2021 spring	3	7	5	1
8	fire	2021 spring	1	11	0	1
9	fire	2021 spring	2	3	1	0
10	fire	2021 spring	0	1	0	0
11	control	2021 spring	2	5	0	2
12	control	2021 spring	2	4	4	1
13	control	2021 spring	3	3	0	0
14	control	2021 spring	2	4	4	0
15	control	2021 spring	1	3	1	0
16	control	2021 spring	1	3	2	2
17	control	2021 spring	1	2	1	0
18	control	2021 spring	4	2	1	0
19	control	2021 spring	4	4	6	1
20	control	2021 spring	3	1	2	0
